# Litter mixing promoted decomposition and altered microbial community in common bean root litter

**DOI:** 10.1186/s12866-023-02871-4

**Published:** 2023-05-23

**Authors:** Linlin Zhang, Jiawei Li, Zhilin Wang, Dinghong Zhang, Hui Liu, Jia Wang, Fengzhi Wu, Xue Wang, Xingang Zhou

**Affiliations:** 1 Department of Horticulture, Northeast Agricultural University, Harbin, China; 2 Northeast Agricultural University Library, Northeast Agricultural University, Harbin, China; 3 Key Laboratory of Biology and Genetic Improvement of Horticultural Crops (Northeast Region), Ministry of Agriculture and Rural Affairs, Northeast Agricultural University, Harbin, China

**Keywords:** Litter mixing, Weight loss, Microbial decomposer, Microbial community abundance, Microbial community composition

## Abstract

**Background:**

Decomposition of plant litter is a key driver of carbon and nutrient cycling in terrestrial ecosystems. Mixing litters of different plant species may alter the decomposition rate, but its effect on the microbial decomposer community in plant litter is not fully understood. Here, we tested the effects of mixing with maize (*Zea mays* L.) and soybean [*Glycine max* (Linn.) *Merr.*] stalk litters on the decomposition and microbial decomposer communities of common bean (*Phaseolus vulgaris* L.) root litter at the early decomposition stage in a litterbag experiment.

**Results:**

Mixing with maize stalk litter, soybean stalk litter, and both of these litters increased the decomposition rate of common bean root litter at 56 day but not 14 day after incubation. Litter mixing also increased the decomposition rate of the whole liter mixture at 56 day after incubation. Amplicon sequencing found that litter mixing altered the composition of bacterial (at 56 day after incubation) and fungal communities (at both 14 and 56 day after incubation) in common bean root litter. Litter mixing increased the abundance and alpha diversity of fungal communities in common bean root litter at 56 day after incubation. Particularly, litter mixing stimulated certain microbial taxa, such as *Fusarium*, *Aspergillus* and *Stachybotrys* spp. In addition, a pot experiment with adding litters in the soil showed that litter mixing promoted growth of common bean seedlings and increased soil nitrogen and phosphorus contents.

**Conclusions:**

This study showed that litter mixing can promote the decomposition rate and cause shifts in microbial decomposer communities, which may positively affect crop growth.

**Supplementary Information:**

The online version contains supplementary material available at 10.1186/s12866-023-02871-4.

## Background

Modern agriculture is usually based on monocropping, which can negatively affect crop production compared with diversified cropping systems, such as cover cropping, intercropping and crop rotation systems [1–3]. Litter decomposition is an important process in regulating the carbon cycle and nutrient dynamics [4, 5]. In diversified cropping systems, plant litter from different species usually mix and decompose together rather than alone [4, 6–8]. Studies have shown that litter mixing may produce a non-additive effect on decomposition [5, 6, 9]. This effect means that the decomposition rate of litter mixture is not the average rate of the component litters, and the decomposition may be increased or decreased due to synergistic or antagonistic interactions [10]. The non-additive effect of litter mixing on decomposition can be explained by several mechanisms, such as: (1) nutrient transfer between litters, i.e., mixing low-quality litter with high-quality litter could increase decomposition rate through transferring nutrients; (2) component species litters with secondary metabolites (such as phenols or tannins) can inhibit the decomposition of litter mixture; (3) changes in micro-environmental conditions, where litter mixing alters the complexity and spatial heterogeneity of the environment, and thus affect decomposition process [4, 6, 11, 12].

Plant litter decomposition is primarily controlled by climate, litter quality (e.g., physical and chemical characteristics of litter), decomposer community and the interactions among these factors [13–15]. Microorganisms, such as bacteria and fungi, are regarded as the main decomposers of litter [16–18]. At the early stage of litter decomposition, bacteria grow rapidly and play a dominant role; whereas in the later stage, the slow growing fungi generally dominate this process [19–21]. After entering soils, specific litter type, with particular morphological and chemical traits, can act an ecological filter to selecting or excluding microbial taxa from the common soil pool [14, 22]. Therefore, litters with differing morphological and chemical traits are usually inhabited by microbial communities with different composition [23, 24]. It is also speculated that litter mixing may stimulate or suppress the growth of certain microbial taxa through special feeding preference for nutrients, or the effects of secondary metabolites and recalcitrant materials [10, 25, 26].

Common bean (*Phaseolus vulgaris* L.) is a vegetable that usually monocropped, especially for the greenhouse production. Cover cropping of soybean (*Glycine max* (Linn.) *Merr.*) and maize (*Zea mays* L.) or incorporating litters of these crops are usually adopted to improve the soil quality and promoted crop growth [27]. Here we investigated the impact of mixing stalks of soybean and maize on the decomposition and microbial community in common bean root litter the early decomposition stage. Since the separation of component species behavior within the litter mixture is a prerequisite to identify the mechanisms by which litter mixing influences decomposition [28], we used double-layer litterbags [29]. Moreover, a pot experiment was performed to evaluate the effects of litter mixing on the growth of common bean. We hypothesized that litter mixing could promote litter decomposition and alter the microbial communities in common bean root litter.

## Results

### Litter weight loss

**Fig. 1:**
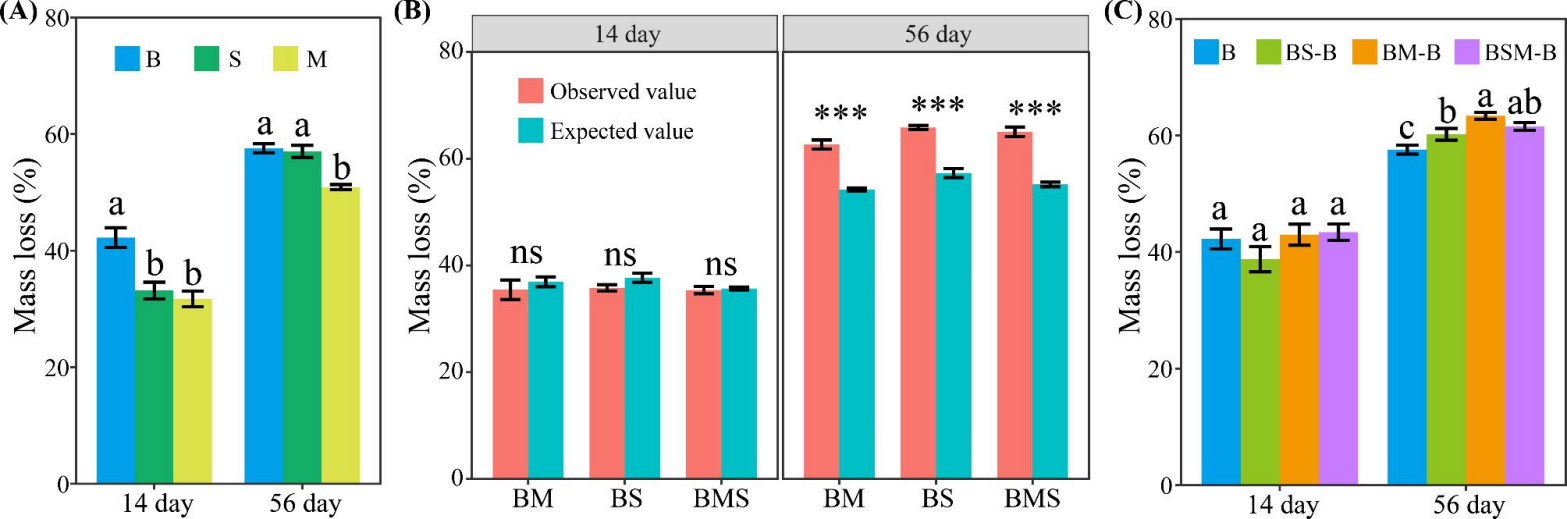
Weight loss of single litters (**A**), the observed and expected weight losses of the whole liter mixture (**B**), and weight loss of common bean root litter in the mixture (**C**). B: common bean root litter, S: soybean stalk litter, M: maize stalk litter. BS, BM, BSM represent common bean root litter mixed with soybean stalk litter, maize stalk litter, and both soybean and maize stalk litters, respectively. –B: common bean root litter in the mixture. ***values were significantly different at *P* < 0.001

The mass loss of common bean root litter was higher than both soybean and maize stalk litters at 14 day after incubation, and was higher than maize stalk litter at 56 day after incubation (Fig. 1A). Moreover, the mass loss of soybean stalk litter was higher than maize stalk litter at 56 day after incubation. Litter mixing increased the decomposition rate of the whole liter mixture at 56 day after incubation but not at 14 day after incubation (*P* < 0.05) (Fig. 1B). Litter mixing at 56 day after incubation, but not at 14 day after incubation, promoted the decomposition of common bean root litter in the litter mixtures, with mixing with maize stalk litter showing the strongest promoting effect (Fig. 1C).

The common bean root litter had higher initial nitrogen, phosphorus and phenol contents, but lower carbon content than soybean and maize stalk litters (nitrogen contents for soybean, maize, and common bean were 7.7, 6.7 and 21.0 g kg^-1^, respectively; phosphorus contents were 1.6, 2.9 and 3.3 g kg^-1^, respectively; phenol contents were 0.19, 0.36 and 0.61 g kg^-1^, respectively; carbon contents were 397, 402 and 365 g kg^-1^, respectively). The calcium content was lower in maize stalk litter (1.4 g kg^-1^) than in soybean and common bean litters (2.8 and 2.4 g kg^-1^, respectively).

### Nitrogen and phosphorus contents in common bean root litter

Litter mixing did not alter nitrogen and phosphorus contents in common bean root litter at 14 day after incubation (Table 1). At 56 day after incubation, mixing with soybean stalk litter increased nitrogen content in common bean root litter, while mixing with maize and both maize and soybean stalk litters decreased nitrogen content in common bean root litter (Table 1). In addition, the weight loss of common bean root litter was significantly correlated with the phosphorus content of common bean root litter at 56 d after incubation (*R*^2^ = 0.514, *P* < 0.05), but not at 14 d after incubation (*R*^2^ = -0.179, *P* > 0.05). The weight loss of common bean root litter was not correlated with the nitrogen content of common bean root litter (*R*^2^ = -0.110, *P* > 0.05; *R*^2^ = -0.222, *P* > 0.05 at 14 and 56 d after incubation, respectively).

**Table 1:** Nitrogen and phosphorus contents in common bean root litter

Content (g kg^− 1^)	Treatments	14 day	56 day
Nitrogen	B	11.30 ± 0.94^a^	11.83 ± 0.28^b^
	BS-B	11.69 ± 0.32^a^	13.53 ± 0.40^a^
	BM-B	11.95 ± 1.13^a^	12.54 ± 0.25^ab^
	BSM-B	11.17 ± 0.71^a^	12.59 ± 0.40^ab^
Phosphorus	B	9.01 ± 0.32^a^	9.72 ± 0.35^c^
	BS-B	9.72 ± 0.55^a^	8.94 ± 0.10^bc^
	BM-B	8.97 ± 0.52^a^	8.42 ± 0.48^ab^
	BSM-B	9.00 ± 0.24^a^	8.04 ± 0.09^a^

B: common bean root litter. BS, BM, BSM represent common bean root litter mixed with soybean stalk litter, maize stalk litter, and both soybean and maize stalk litters, respectively. –B: common bean root litter in the mixture. Different letters indicate significant difference between treatments (Tukey’s HSD test, *P* < 0.05)

### Microbial community abundances in common bean root litter

**Fig. 2:**
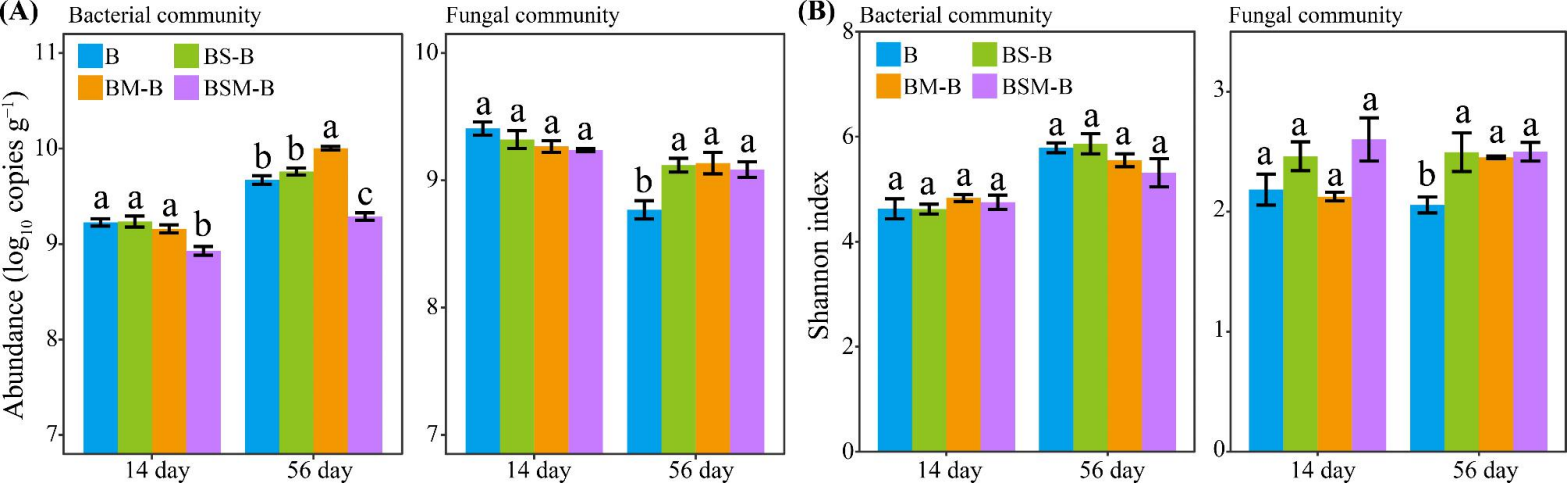
Abundances (**A**) and alpha diversities (**B**) of bacterial and fungal communities in common bean root litter. B: common bean root litter. BS, BM, BSM represent common bean root litter mixed with soybean stalk litter, maize stalk litter, and both soybean and maize stalk litters, respectively. –B: common bean root litter in the mixture. Different letters indicate significant difference between treatments (Tukey’s HSD test, *P* < 0.05)

At both 14 and 56 day after incubation, mixing with both maize and soybean stalk litters decreased the bacterial abundance in common bean root litter (Fig. 2A). Moreover, mixing with maize stalk litter increased bacterial abundance in common bean root litter at 56 day after incubation. The fungal community abundance in common bean root litter did not differ among treatments at 14 day after incubation, however, the fungal community abundance in common bean root litter was prompted by all litter mixtures at 56 day after incubation (Fig. 2A).

### Microbial community diversity and composition in common bean root litter

**Fig. 3:**
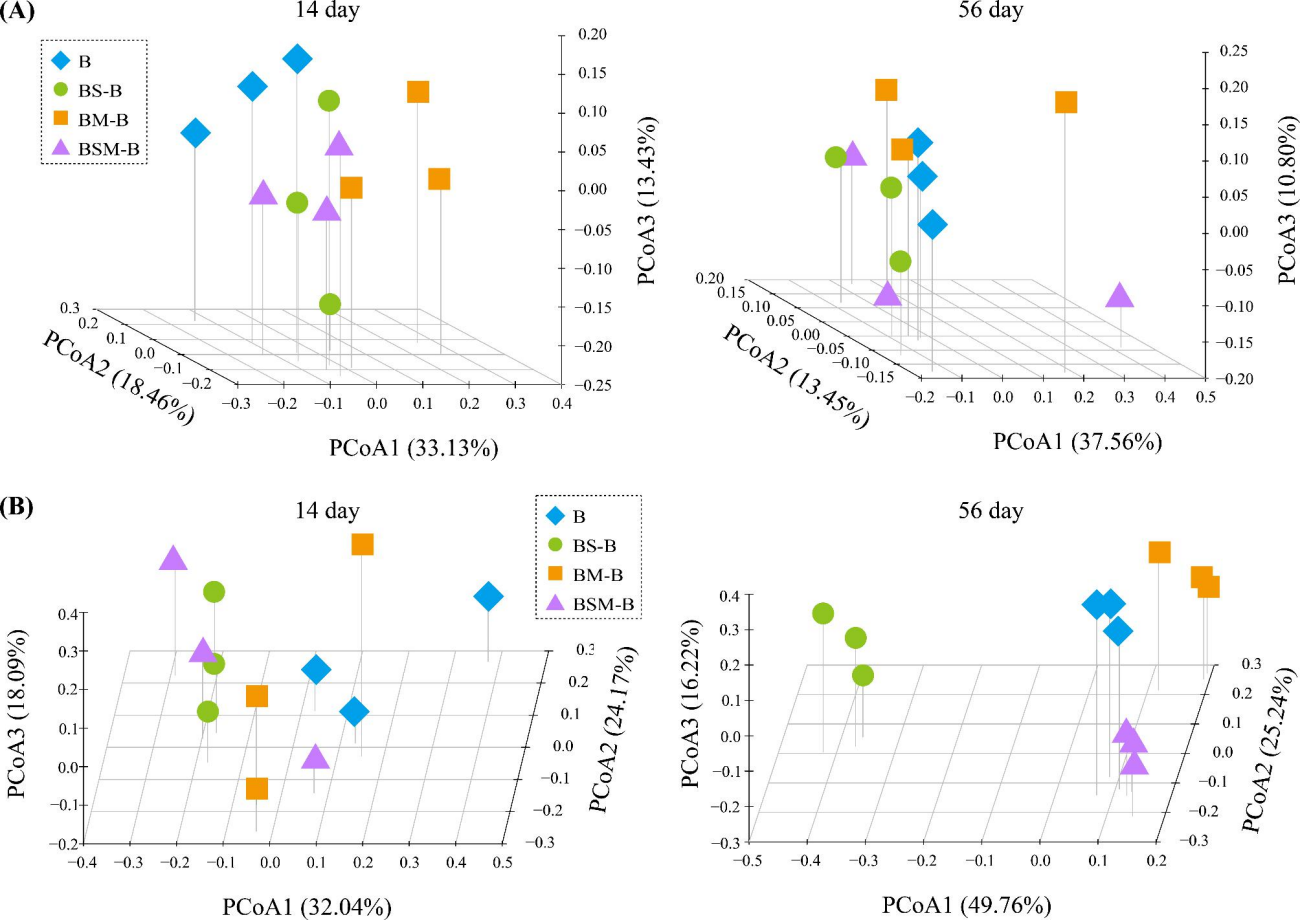
Principal coordinate analysis (PCoA) plots of bacterial (**A**) and fungal (**B**) communities at each sampling time. B: common bean root litter. BS, BM, BSM represent common bean root litter mixed with soybean litter, maize litter, and both soybean and maize litter, respectively. –B: common bean root litter in the mixture

Illumina Miseq sequencing yielded a total of 1,076,600 quality 16 S rDNA sequences and 1,243,504 quality ITS sequences. Litter mixing did not affect the alpha diversity (i.e., the Shannon index) bacterial community in common bean root litter (Fig. 2B). However, all litter mixtures increased the Shannon index of fungal community in common bean root litter at 56 day after incubation. For microbial beta diversities, PCoA analysis showed that bacterial and fungal communities in common bean root litter differed between the two sampling times (Fig. S1). PERMANOVA analysis confirmed that sampling period had significant effects on bacterial and fungal community beta diversities in common bean root litter (*R*^2^ = 0.489, *P* < 0.001; *R*^2^ = 0.383, *P* < 0.001, respectively). Litter mixing also altered bacterial community beta diversity in common bean root litter at 14 day after incubation but not at 56 day after incubation (PERMANOVA, *R*^2^ = 0.385, *P* < 0.001; *R*^2^ = 0.343, *P* > 0.05, respectively) (Fig. 3A). Moreover, litter mixing also altered fungal community beta diversity in common bean root litter at both 14 and 56 day after incubation (PERMANOVA, *R*^2^ = 0.536, *P* < 0.001; *R*^2^ = 0.858, *P* < 0.001, respectively) (Fig. 3B).

**Fig. 4:**
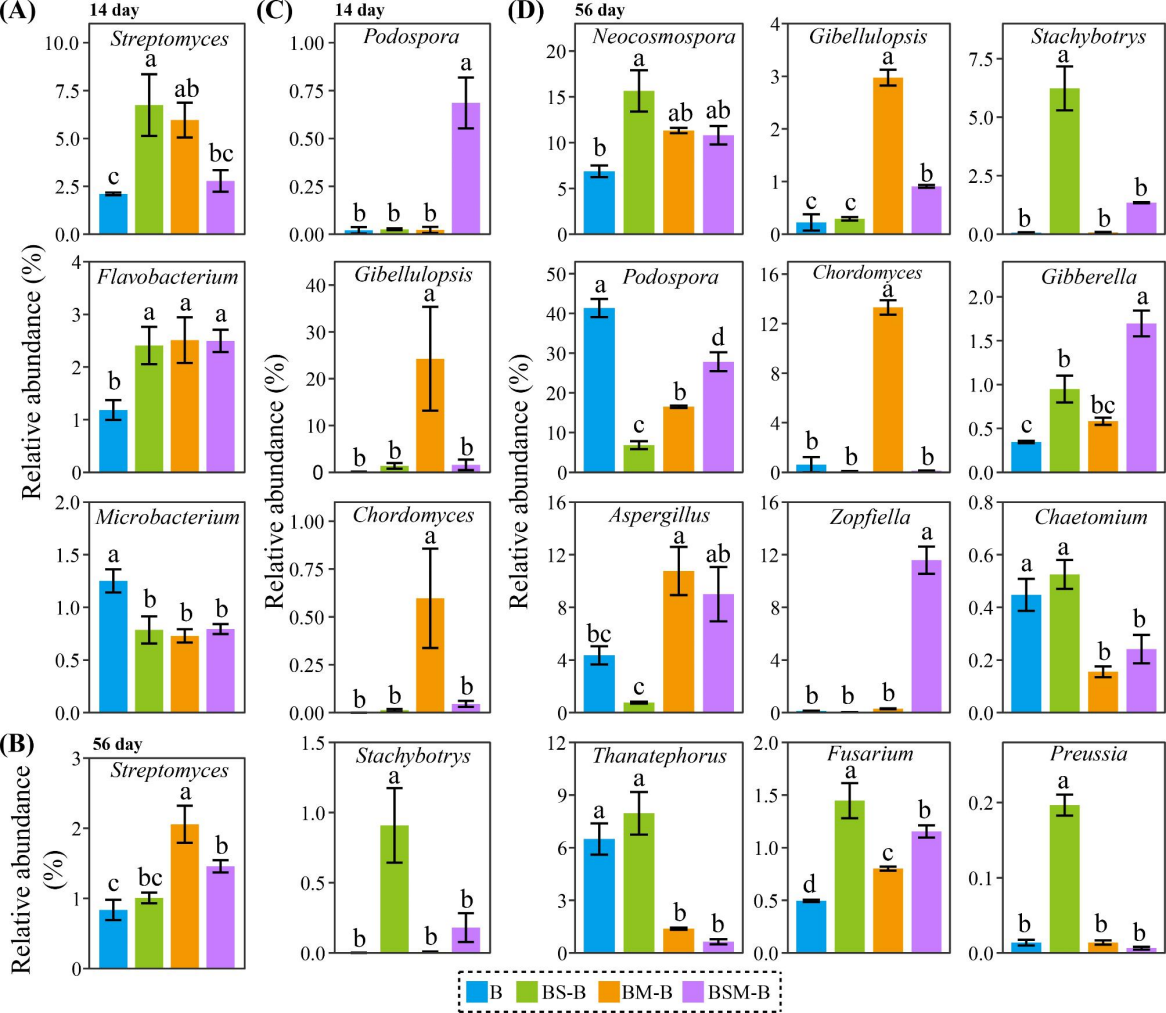
Bacterial (**A, B**) and fungal (**C, D**) genera altered by litter mixing at 14 and 56 day after incubation. B: common bean root litter. BS, BM, BSM represent common bean root litter mixed with soybean stalk litter, maize stalk litter, and both soybean and maize stalk litter, respectively. –B: common bean root litter in the mixture. Different letters indicate significant difference between treatments (Tukey’s HSD test, *P* < 0.05)

For bacterial community in common bean root litter, about 87.20% of the sequences belong to phyla Proteobacteria, Firmicutes, Bacteroidetes and Actinobacteria (Fig. S2A). The dominant bacterial classes (relative abundance > 5% across all samples) were Alphaproteobacteria, Gammaproteobacteria, Deltaproteobacteria, Actinobacteria, Bacilli and Sphingobacteriia, which account for 76.98% of all the bacterial sequences (Fig. S2B). The relative abundances of phylum Actinobacteria and class Actinobacteria in common bean root litter were increased when mixed with maize stalk litter, while these of phylum Saccharibacteria and class Saccharibacteria norank were decreased when mixed with both soybean and maize stalk litters at 56 day after incubation (Fig. S2). At the genus level, mixing with soybean or maize stalk litter increased the relative abundances of *Streptomyces* and *Flavobacterium* spp. while decreased that of *Microbacterium* sp. at 14 day after incubation (Fig. 4A). Mixing with both soybean and maize stalk litters increased the relative abundance of *Flavobacterium* sp. while decreased that of *Microbacterium* sp. at 14 day after incubation. Mixing with maize stalk litter, and both soybean and maize stalk litters increased the relative abundance of *Streptomyces* sp. at 56 day after incubation (Fig. 4B). Indicator species analysis identified 184 bacterial OTUs that were altered by litter mixing (differential OTUs) (Fig. S4). These differential OTUs were mainly classified as Proteobacteria, Bacteroidetes, Actinobacteria and Firmicutes. For example, the relative abundance of *Microbacterium* sp. OTU698 was found to be the lowest in the common bean root litter treatment. The relative abundances of *Flavobacterium* sp. OTU54 and OTU910 were stimulated by mixing with both soybean and maize stalk litters and maize stalk litter respectively.

**Fig. 5:**
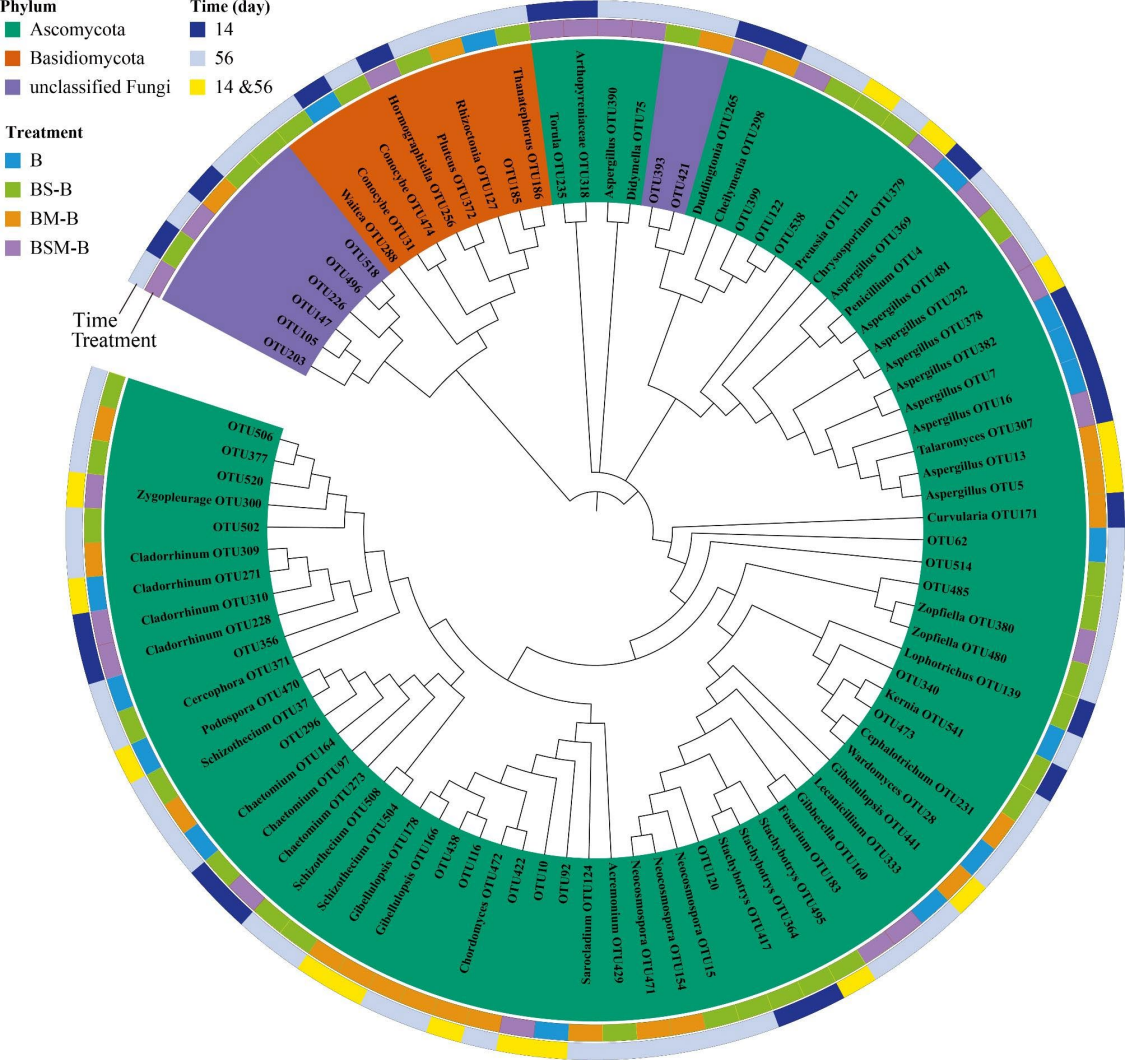
Dendrogram showing fungal OTUs altered by litter mixing. The first strip indicates the phylum-level affiliation of each. The second strip indicates in which treatment each differential OTU is enriched. The third strip indicates sampling time. The size of each circle indicates the relative abundance of each differential OTU. B: common bean root litter. BS, BM, BSM represent common bean root litter mixed with soybean stalk litter, maize stalk litter, and both soybean and maize stalk litter, respectively. –B: common bean root litter in the mixture

Dominant fungal phyla in common bean root litter were Ascomycota and Basidiomycota, which accounted for 99.33% of the total fungal sequences (Fig. S3A). The relative abundance of Basidiomycota was increased while that of Ascomycota was decreased by mixing with maize stalk litter and both soybean and maize stalk litters at 56 day after incubation (Fig. S3A). At the class level, Sordariomycetes, Agaricomycetes and Eurotiomycetes were the dominant fungal classes (average relative abundance > 1% across all samples) (Fig. S3). At 14 day after incubation, mixing with maize litter and both soybean and maize stalk litters increased the relative abundance of Sordariomycetes (Fig. S3B). At 56 day after incubation, mixing with maize stalk litter and both soybean and maize stalk litters decreased the relative abundance of Agaricomycetes while increased that of Eurotiomycetes. At the genus level, mixing with both soybean and maize stalk litters increased *Podospora* sp., mixing with maize stalk litter increased *Gibellulopsis* and *Chordomyces* spp., and mixing with soybean stalk litter increased *Stachybotrys* sp. at 14 day after incubation (Fig. 4C). At 56 day after incubation, mixing with soybean stalk litter increased *Neocosmospora*, *Stachybotrys*, *Gibberella*, *Preussia* spp., mixing with maize stalk litter increased *Gibellulopsis*, *Chordomyces*, and *Aspergillus* spp., while mixing with both soybean and maize stalk litters increased *Gibberella* and *Zopfiella* spp. (Fig. 4D). Worth noting, all litter mixing treatments stimulated *Fusarium* sp. in common bean root litter. Indicator species analysis identified 90 fungal OTUs that were altered by litter mixing (Fig. 5). For example, *Fusarium* sp. OTU183, *Gibberella* sp. OTU160, *Zopfiella* sp. OTU380, *Aspergillus* sp. OTU292 and OTU390 were stimulated by mixing with both soybean and maize stalk litters at 56 day after incubation.

### Common bean seedling growth and soil nutrient contents

**Fig. 6:**
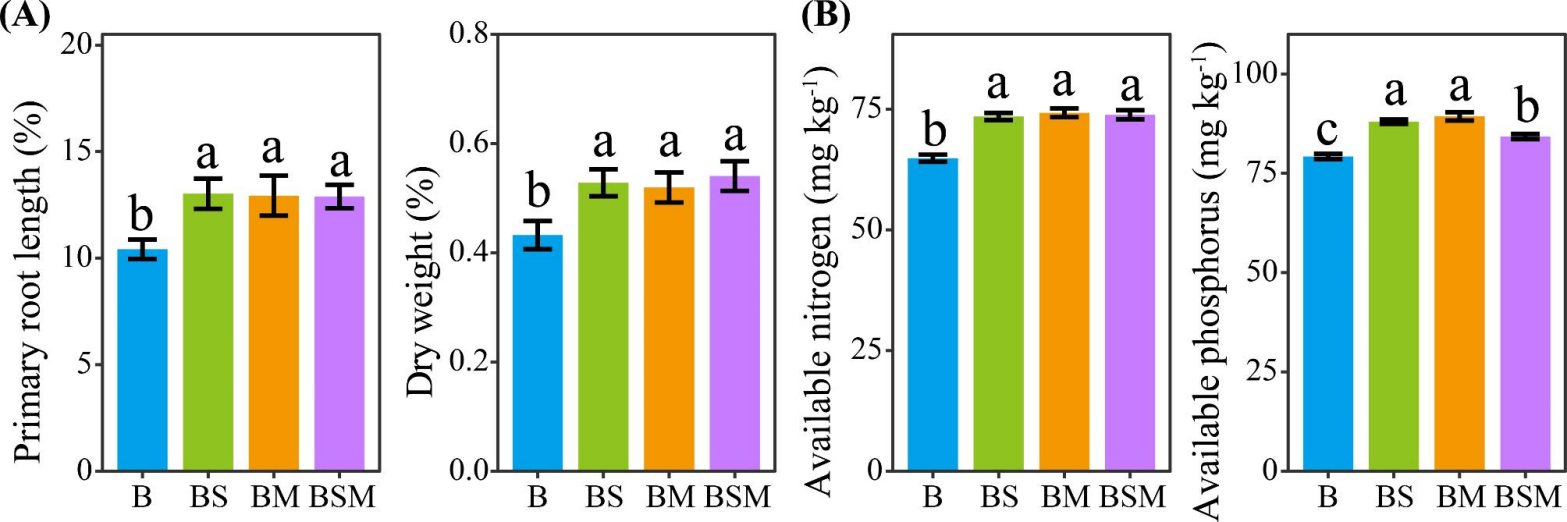
Common bean seedling growth (**A**) and soil nutrient contents (**B**) in the pot experiment (mean ± SE). B: common bean root litter. BS, BM, BSM represent common bean root litter mixed with soybean stalk litter, maize stalk litter, and both soybean and maize stalk litter, respectively. Different letters indicate significant difference between treatments (Tukey’s HSD test, *P* < 0.05)

The primary root length and dry weight of common bean seedlings grown in soils with litter mixtures were higher than that in soils with only common bean root litter (Fig. 6A, Fig. S5). Common bean seedling dry weight was increased by 22.19%, 20.20% and 25.04% in the treatment of common bean root litter mixed with soybean stalk litter, maize stalk litter, or both soybean and maize stalk litter, respectively, as compared with the treatment of common bean root litter. Moreover, treatments with litter mixtures had higher soil available nitrogen and phosphorus contents than the treatment with only common bean root litter (Fig. 6B).

## Discussion

Previous studies found that litter mixing usually generate synergistic non-additive effects more frequently than antagonistic non-additive effects on litter decomposition [6, 9, 12]. Here, we also found that litter mixing promoted the decomposition of the whole mixture at 56 day after incubation. Separation of common bean root litter from other litters in mixture using double-layer litterbags [29] allowed us to identify the impact of litter mixing on the decomposition of a component litter species in the mixture. The mass loss of common bean root litter was accelerated when mixed with soybean stalk litter, maize stalk litter, or both soybean and maize stalk litters, which supported our first hypothesis that litter mixing could promote the decomposition of common bean root litter. Since the common bean root litter had higher nitrogen and phosphorus contents than both soybean and maize stalk litters, the observed enhanced decomposition of common bean root litter in the mixture might be due to changes in micro-environmental conditions, such as increasing in the habitat heterogeneity, but not to transfer of nutrients [11, 12, 30, 31].

Plant litter type is an important regulator of microbial decomposer communities [32, 33]. Thus, we focused on the microbial communities in one component litter species in the litter mixture, but rather than the microbial communities of the whole mixture. In this study, sampling time was an important factor affecting the assembly of bacterial and fungal communities on common bean root litter, which supported previous studies [20, 34]. This succession of microbial communities during decomposition has been proposed to be driven by changes in resource availability [17, 20, 35]. In line with previous observations [10, 26, 36], we found litter mixing altered the diversities and abundances of bacterial and fungal communities on common bean root litter, which validated our hypothesis. Particularly, litter mixing increased fungal community abundance and alpha diversity. A microbial community with higher diversity can generally support a higher level of ecosystem functioning, such as biomass production and decomposition, through both facilitative interactions and resource partitioning among microbial species [37–39]. Moreover, compared with bacteria, fungi are relatively more important in decomposing of low quality litter [21, 40, 41]. Therefore, the enhanced decomposition of common bean root litter might be linked to the increased fungal community alpha diversity and abundance. We also found that fungal and bacterial communities respond differently to litter mixing. For example, litter mixing increased the alpha diversity of fungal community but not the bacterial community at 56 day after incubation. These results suggested that fungal and bacterial communities play different roles in decomposition [4, 20, 42]. In the present study, we only focused on the microbial decomposers and litterbags with 250 μm nylon mesh were used. Therefore, the function of the large-body decomposers, such as detritivore fauna, should be further evaluated.

Litter mixing was shown to positively and negatively affect the abundances of specific bacterial and fungal taxa in common bean root litter, thereby altering the compositions of bacterial and fungal communities. Plant litter is an oligotrophic habitat and a relatively narrow group of microorganism are able to degrade complex recalcitrant compounds in the litter (e.g., cellulose, hemicellulose and lignin) [17, 43, 44]. Particularly, we found that litter mixing stimulated some microbial taxa such as *Gibellulopsis*, *Stachybotrys*, *Gibberella*, *Aspergillus*, *Fusarium* and *Preussia* spp., which have been reported to have decomposing abilities of recalcitrant compounds [45–50]. A recent study also found that *Fusarium* sp. in root litter of tomato (*Solanum lycopersicum* L.) was stimulated by litter mixing, and cultured representative isolates of this taxon was shown to have decomposing ability [6]. Therefore, the observed variation in microbial community composition, especially the increases in specific microbial taxa, might result in rapid decomposition of common bean root litter. Isolating and testing the litter-decomposing abilities of these stimulated microbial taxa are necessary to improve our understanding of the role of microbial decomposer community in the litter mixing-effect.

In agriculture, cover cropping and using organic amendments are usually used to improve soil fertility and promote crop growth [3, 7, 51]. Accelerated litter decomposition may reduce the negative effects of autotoxins released from plant root litter [52, 53]. Our pot experiment found that litter mixing stimulated common bean seedling growth and soil nutrient contents. It has been reported that the accelerated litter decomposition can promote recycling of elements in the soil [24, 54]. The increased soil nutrients may be directly released from the decomposed plant litters. Meanwhile, inputs of exogenous substrate, such as plant litter, may stimulate the activity of microorganisms that decompose soil organic matter and release plant available nutrients (i.e., the priming effect) [55–57]. Our results suggested that plant litter mixing could accelerate decomposition and recycling of elements, which further generated positive effects on plant growth. The growth period of common bean is about two to three months. Therefore, we measured the decomposition rate of the whole liter mixture for a relative short time period. Here, we found that litter mixing altered the decomposition of the whole mixture and common bean root litter at 56 day but not at 14 day after incubation. This observation is consistent with previous finding that litter mixing-effect could vary at different decomposition stages [58]. Nevertheless, further experiments are warranted to assess the long-term effects of the litter mixing-effect.

## Conclusion

We found that the decomposition of common bean root litter could be promoted by mixing with other crop litter (i.e., soybean and maize stalk litters) the early decomposition stage. Litter mixing generated a synergistic effect on the decomposition of the litter mixture. Moreover, mixing common bean root litter with other crops litters altered the diversities and compositions of microbial decomposer communities and increased the relative abundances of certain taxa as potential decomposers. Litter mixing also promoted the growth of common bean seedlings and increased soil nitrogen and phosphorus contents. Our study highlights that it is possible to manipulate litter diversity and certain microbial taxa to regulate litter decomposition, and thus enhance the sustainability of agroecosystems.

## Methods

### Soil and plant litter preparation

Soil was taken from the upper layer (0–15 cm) in a greenhouse that had been continuously cropped with common bean for more than 10 years in the experimental station of Northeast Agricultural University, Harbin, China (45°41’N, 126°37’E). The basic properties of the soil were: organic matter, 46.37 g kg^-1^; available phosphorus, 80.18 mg kg^-1^; available nitrogen, 64.40 mg kg^-1^; pH (1:5 w/v), 7.23; and electrical conductivity (1:5 w/v), 0.32. After sieving (2 mm) to remove large stones and visible roots, soil samples were thoroughly mixed, and then pre-incubated at 25 °C for five days with water holding capacity kept at 60% before use.

Stalks of soybean and maize, and root litter of common bean were collected after the harvest of each crop in autumn 2017. Stalks of maize and soybean were cut into 1 ~ 2 cm length pieces. Roots (≤ 2 mm diameter) of common bean were washed with tap water to remove the soil particles, oven-dried at 80 °C to constant weight, and cut into small pieces (1 ~ 2 cm length). A portion of these dried litters were grounded, and digested with sulfuric acid to measure the chemical properties, including total nitrogen, phosphorus, calcium, and polyphenol contents.

### Litterbag experiment

Double-layer litterbags with two adjacent compartments were used in order to separate different litter species at the time of harvest [6, 29]. Double-layer litterbags (6 × 9 cm) used were with outside of 250 μm nylon mesh and inside of 1 mm nylon mesh [6]. There were six treatments consisting of: both compartments filled with (1) soybean stalk litter (S), (2) maize stalk litter (M), or (3) common bean root litter (B), respectively; one compartment filled with common bean root litter while the other compartment with (4) soybean stalk litter (BS), (5) maize stalk litter (BM), or (6) both maize and soybean stalk litters (BMS), respectively. Each litterbag contained a total of 1.5 g of single or mixed litters (equal w/w proportion). Each treatment had 24 replicates. One litterbag was put in one plastic bottle filled with 150 g of soil and buried horizontally 5-cm below the soil surface. Soil moisture was maintained at 60% of the water holding capacity. Litterbags were harvested after 14 d and 56 d of incubation, respectively. At each sampling time, four replicates in each treatment were used to measure weight loss and the total nitrogen, phosphorus contents. After opened, soil particles on litters were carefully removed by washing with tap water over a sieve (200 μm mesh) to ensure that all the litter was retained. Meanwhile, another three replicates in each treatment from each treatment containing common bean were used to collect common bean root litter to analysis of microbial community. Common bean root litter was carefully cleaned with a fine brush to remove adhesive soil and stored at -80 °C before DNA extraction.

### DNA extraction and qPCR analysis

Total DNA was extracted from 0.25 g of common bean root litter with the PowerSoil DNA Isolation Kit (MO BIO laboratories, Carlsbad, USA) according to the manufacturers’ instruction. Abundances of bacterial and fungal communities in common bean root litter were determined by SYBR Green qPCR on anIQ5 Real-time PCR system (Bio-Rad Lab, LA, USA) using primer sets F338/R518 [59] and FITS1/RITS4 [60], respectively. The PCR reaction mixture contained 9 µL of 2 × Real SYBR Mixture (TianGen, Beijing, China), 0.2 µL of 10 µM forward and reverse primers (each), 8.1 µL of sterilized water, and 2.5 µL of DNA. The PCR protocols were: 94 °C for 3 min (94 °C for 5 min for fungi); followed by 32 cycles at 94 °C for 45 s for bacteria (24 cycles at 94 °C for 1 min for fungi), 67.4 °C for 45 s for bacteria (58 °C for 1 min for fungi) and 72 °C for 45 s for bacteria and fungi respectively; and a final elongation at 72 °C for 10 min. Standard curves were created with 10-fold dilution series of plasmids containing the target genes. Sterile water was used as negative control. The threshold cycle (Ct) values obtained for each sample were compared with the standard curve to determine the initial copy number of the target genes.

### Illumina Miseq sequencing and data processing

The compositions of bacterial and fungal communities in common bean root litter were analyzed with Illumina MiSeq sequencing. Primer sets of F338/R806 [61] and ITS1F/ITS2R [60, 62] were used to amplify V3-V4 regions of the bacterial 16 S rDNA and the ITS1 regions of the fungal rDNA, respectively. Both the forward and reverse primers also had a 6-bp barcode unique to each sample, which were used to permit multiplexing of samples. Each DNA sample was independently amplified thrice, and the products of the triplicate PCR reactions were pooled and purified. The mixture was then paired-end sequenced (2 × 300) on an Illumina Miseq platform.

As described previously [2, 63], raw sequence reads obtained from MiSeq sequencing were de-multiplexed and quality filtered through FLASH with following process: (1) truncate the low quality fragments of sequences; (2) cluster sequences at 97% similarity to yield operational taxonomic units through UPARSE [64]; (3) classify the effective sequence of each OTU obtained through the SILVA 132 (bacteria, https://www.arb-silva.de/) and Unite (fungi, http://unite.ut.ee) databases; (4) identify and remove the chimeric sequences through UCHIME in QIIME (http://qiime.org/).

### Pot experiment

A pot experiment was conducted to evaluate the effects of mixing litters on the growth of common bean. After sieving (2 mm), 300 g of soils were filled into pots (8 × 8 cm) and mixed with 3 g of different litters. There were four treatments, (1) only common bean root litter, mixture of common bean root litter and (2) soybean litter, (3) maize stalk litter, or (4) both soybean and maize stalk litters. All litters used were ground and sieved (2 mm) before mixing with the soil. The soil water content was held at about 60% of water holding capacity. Fifteen days later, germinated common bean seeds were planted in pots (one seed per pot). There were nine pots per treatment. All pots were maintained in a greenhouse (day and night temperature respectively of 28 °C and 20 °C, relative humidity of 60–80%, 16-h light/8-h dark cycle). Common bean seedlings were harvested 20 days after planting, and dry biomass and primary root length were measured using a ruler. Meanwhile, bulk soils were sampled to measure available nitrogen and phosphorus contents.

### Litter and soil chemical analysis

Harvested litters were oven-dried at 80 °C to a constant weight and milled (2 mm mesh). Total nitrogen content was measured by Kjeldahl distillation after digesting the plant material with sulfuric acid [65, 66]. Total phosphorus content was determined colorimetrically using the molybdenum blue method after digesting the plant material with sulfuric acid and hydrogen peroxide [67, 68]. The calcium content was evaluated by the complexometric titration method [69]. Total polyphenol content was determined by the Folin-Ciocalteu method by using gallic acid as the standard [66, 70]. Total carbon content was measured with a FlashSmart™ elemental analyzer (ThermoFisher Scientific, Waltham, USA). For soil available nitrogen (nitrate- and ammonium-nitrogen) and phosphorus, soil was extracted with 2 M potassium chloride and 0.5 M sodium bicarbonate, respectively and phosphorus, nitrogen contents in these extracts were determined with a San + + continuous flow analyzer (SKALAR, Breda, Netherlands).

### Statistical analysis

Bacterial and fungal sequences of all samples were rarefied to the minimum number of sequence (28,598 and 31,363 sequences, respectively) per sample. Bacterial and fungal alpha diversity were calculated as the Shannon diversity indices. Principal coordinates analysis (PCoA) based on the Bray-Curtis distance dissimilarity was used to visualize the differences in the compositions of bacterial and fungal communities. Permutational multivariate ANOVA (PERMANOVA) was used to test the effect of decomposition time and litter mixin on microbial community compositions with the Bray-Curtis distance and 9999 random permutations. To test whether a single OTU was associated with a certain treatment, we conducted species indicator analysis with “indicspecies” package in R [71]. A neighbor-joining tree was constructed and drawn in MEGA based on representative sequences for each differently enriched OTU in treatments, and displayed using iTOL (https://itol.embl.de/).

The weight loss of plant litter was calculated as the difference between initial weight and remaining weight at each sampling time. All data were checked for variance normality, heterogeneity and were log-transformed to satisfy the assumption of normality before statistical analysis. Analysis of variance (ANOVA) followed by Tukey’s HSD was used to compare the difference among treatments, *p* < 0.05 was considered as statistical significance.

## Electronic supplementary material

Below is the link to the electronic supplementary material.

Supplementary Material 1

## Data Availability

Sequencing data has been deposited in the Sequence Read Archive at NCBI with the accession numbers PRJNA675145.
