# Supplementary material for: Litter mixing promoted decomposition and altered microbial community in common bean root litter
Source: BMC Microbiol. 2023 May 23;23:148. doi: 10.1186/s12866-023-02871-4 (PMC10204263; doi:10.1186/s12866-023-02871-4)
Supplement: Supplementary file 1 — Supplementary Material 1 [file 12866_2023_2871_MOESM1_ESM.docx]

**Supplementary Materials:**


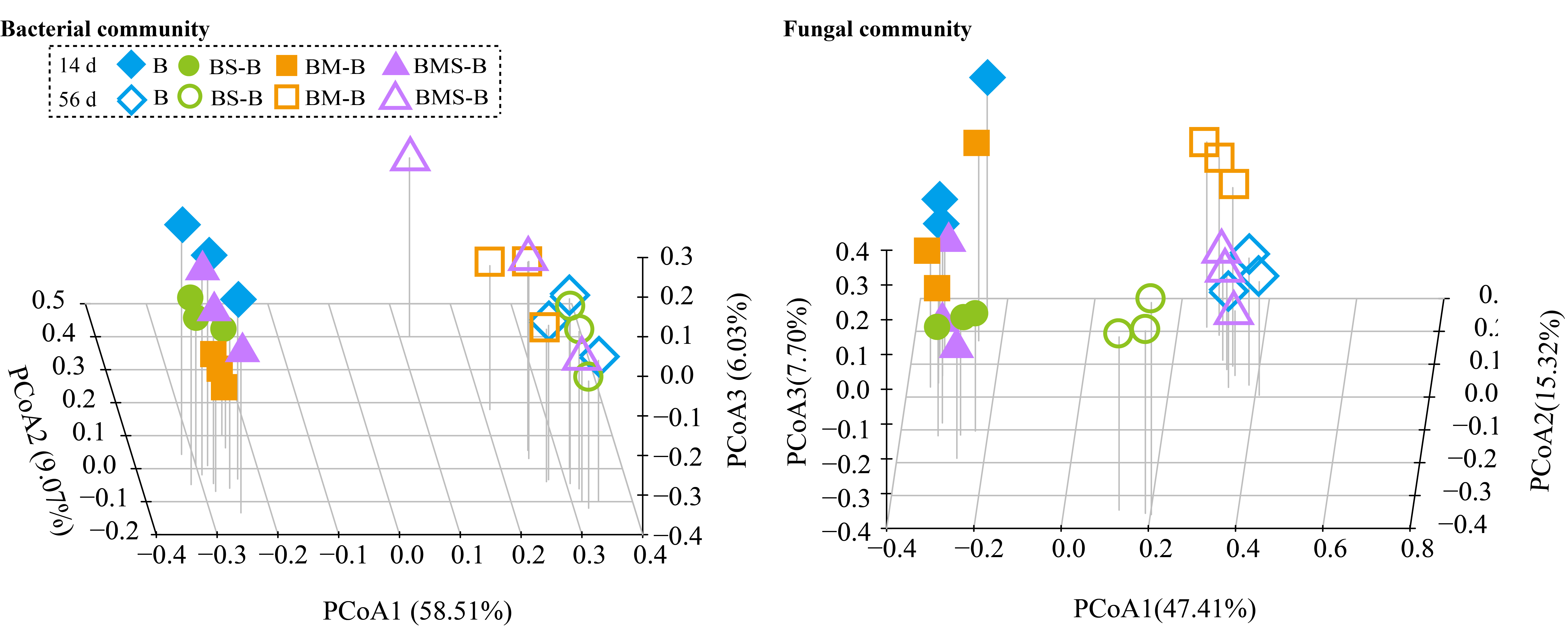


**Fig. S1** Principal coordinate analysis (PCoA) plots of bacterial and fungal communities. B: common bean root litter. BS, BM, BSM represent common bean root litter mixed with soybean stalk litter, maize stalk litter, and both soybean and maize stalk litters, respectively. –B: common bean root litter in the mixtures.


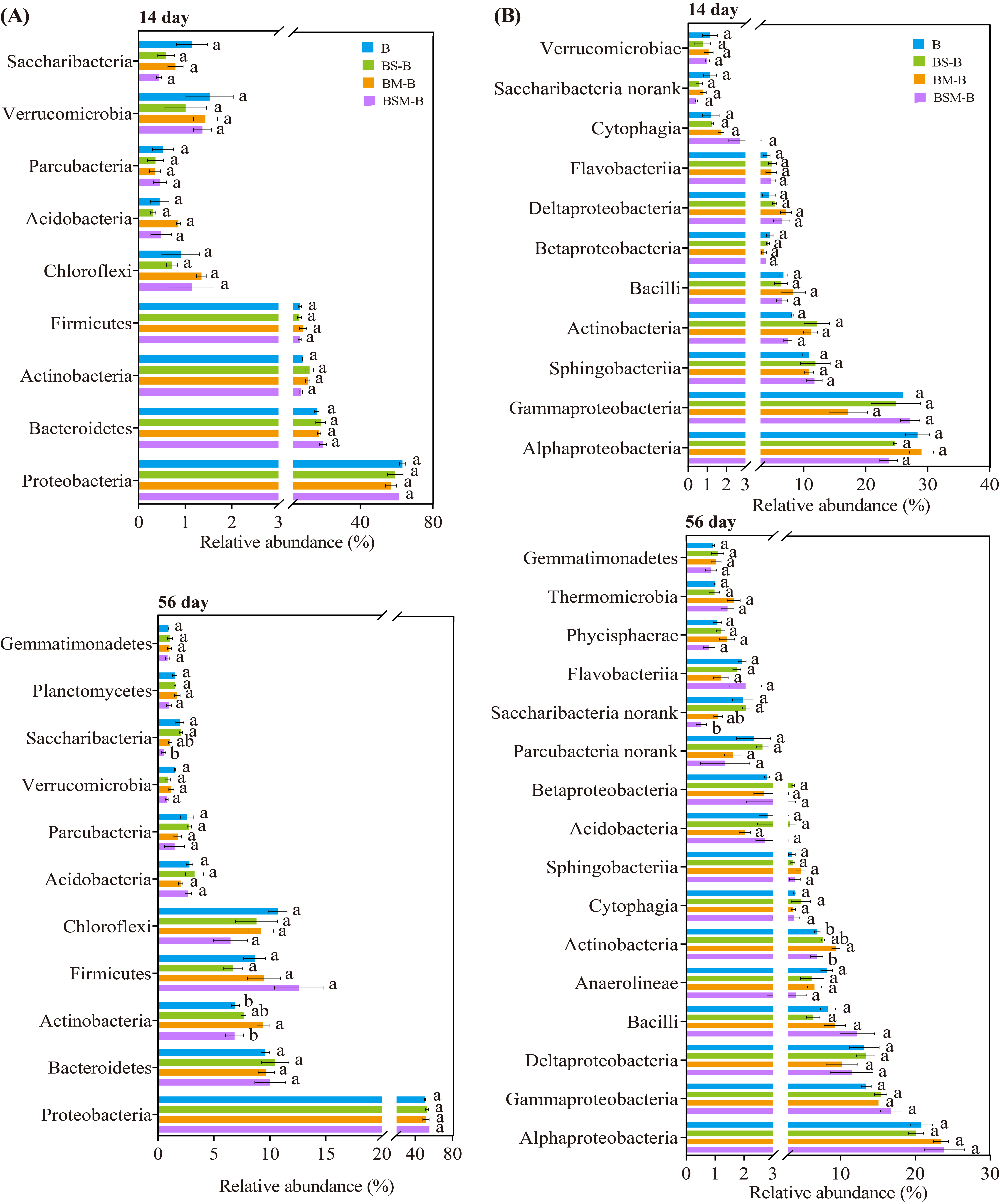


**Fig. S2** Relative abundances of main bacterial phyla (A) and classes (B) in common bean root litter. Bacterial phyla and classes with average relative abundances >1% in at least one treatment were shown (mean±SE). B: common bean root litter. BS, BM, BSM represent common bean root litter mixed with soybean stalk litter, maize stalk litter, and both soybean and maize stalk litters, respectively. –B: common bean root litter in the mixtures. Different letters indicate statistically significant differences among treatments (Tukey’s HSD test, *p* < 0.05).


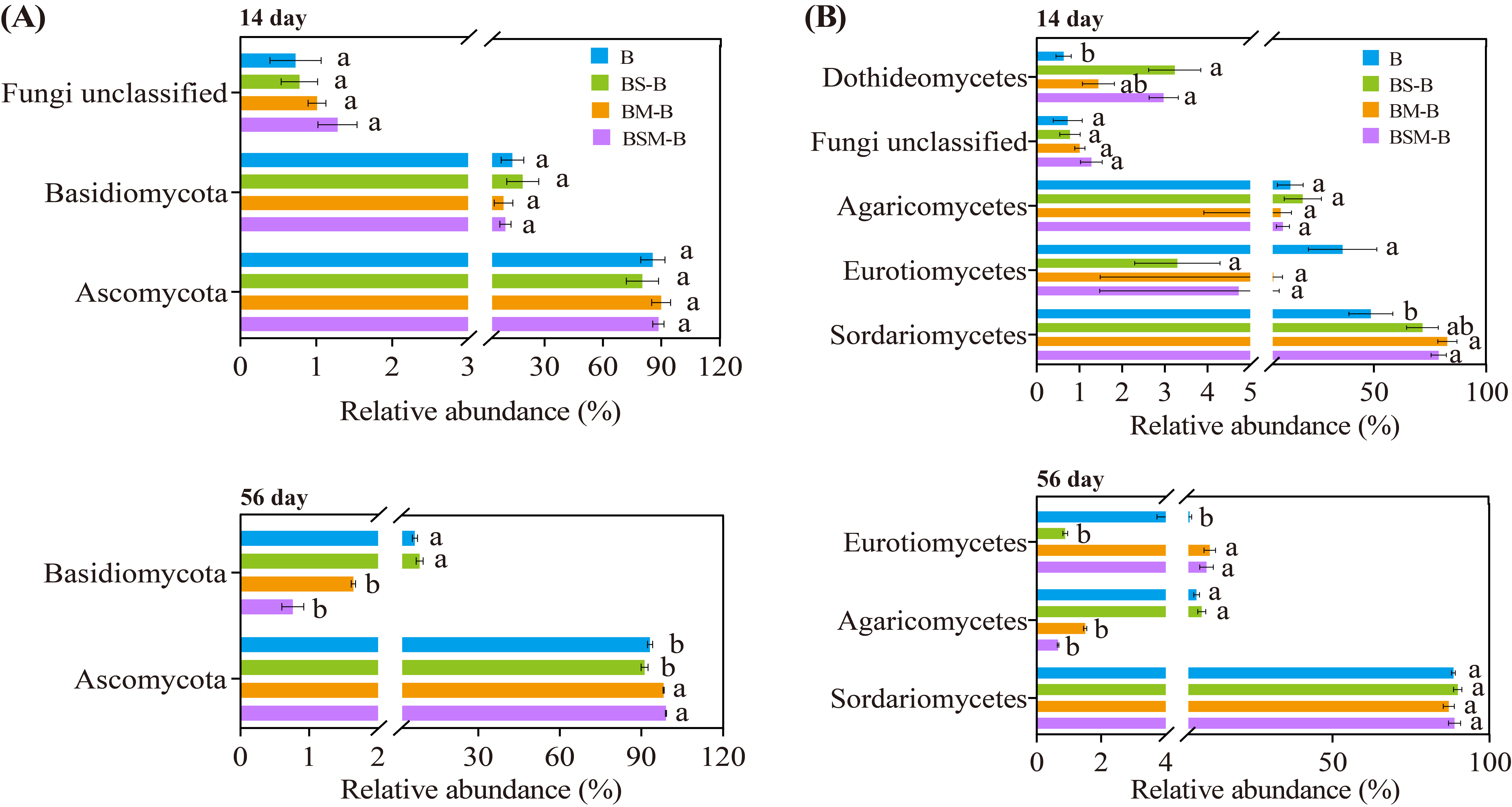


**Fig. S3** Relative abundances of main fungal phyla (A) and classes (B) in common bean root litter. Fungal phyla and classes with average relative abundances >1% in at least one treatment were shown (mean±SE). B: common bean root litter. BM, BS, BMS represent common bean root litter mixed with maize stalk litter, soybean stalk litter, and both maize and soybean stalk litters, respectively. –B: common bean root litter in the mixtures. Different letters indicate statistically significant differences among treatments (Tukey’s HSD test, *p* < 0.05).


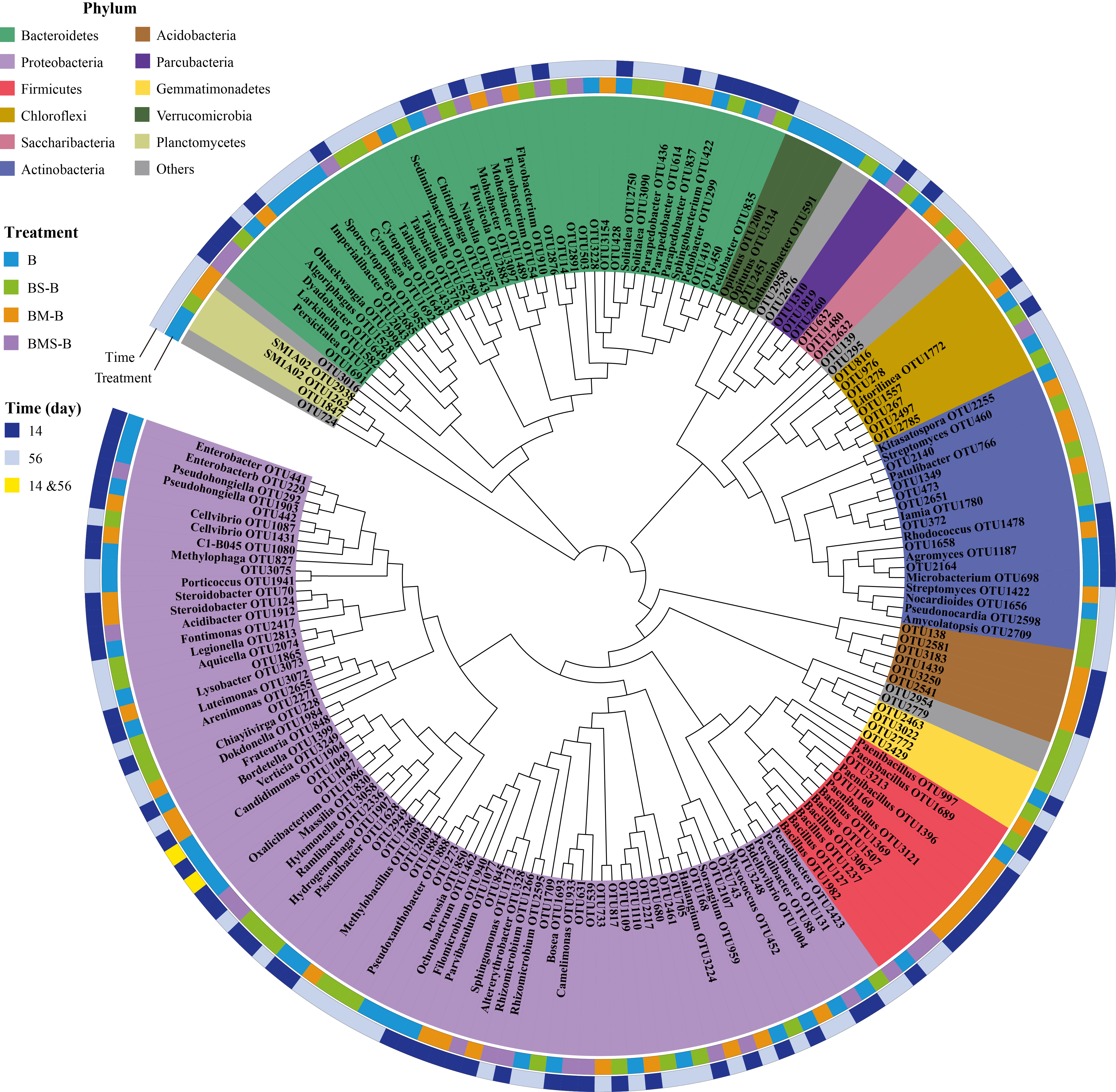


**Fig. S4** Dendrogram showing bacterial OTUs altered by litter mixing. The first strip indicates the phylum-level affiliation of each. The second strip indicates in which treatment each differential OTU is enriched. The third strip indicates sampling time. B: common bean root litter. BS, BM, BSM represent common bean root litter mixed with soybean stalk litter, maize stalk litter, and both soybean and maize stalk litters, respectively. –B: common bean root litter in the mixture.

**Fig. S5** The photo showing the growth of common bean seedlings in each treatment. B: common bean root litter. BS, BM, BSM represent common bean root litter mixed with soybean stalk litter, maize stalk litter, and both soybean and maize stalk litters, respectively.
